# Synergistic potential of yeast, seaweed, and green tea extracts for reducing the application of NPK fertilizers in *Duranta repens* L. fertilization

**DOI:** 10.3389/fpls.2026.1901316

**Published:** 2026-09-07

**Authors:** El-Sayed M. El-Mahrouk, Hayam M. A. Ebrahim, Huda G. Mahmoud, Ahmed M. El-Tarawy, Khaled Abdelaal, Salim A. Ali, Mohammad Fikry, Muhammad Munir, Babiker M. Abdel-Banat, Zafar Iqbal, Alhosein Hamada, Mohamed A. A. Ahmed

**Affiliations:** 1 Horticulture Department, Faculty of Agriculture, Kafrelsheikh University, Kafrelsheihk, Egypt; 2 Agricultural Research Center (ARC), Horticulture Research Institute Alex. Branch (Sabahia), Alexandria, Egypt; 3 Agricultural Botany Department, Faculty of Agriculture, Kafrelsheikh University, Kafrelsheihk, Egypt; 4 Date Palm Research Center of Excellence, King Faisal University, Al-Ahsa, Saudi Arabia; 5 Central Laboratories, King Faisal University, Al-Ahsa, Saudi Arabia; 6 Arid Land Agriculture, College of Agricultural and Food Sciences, King Faisal University, Al-Ahsa, Saudi Arabia; 7 Plant Production Department (Horticulture - Medicinal and Aromatic Plants), Faculty of Agriculture (Saba Basha), Alexandria University, Alexandria, Egypt

**Keywords:** chemical fertilizer, *Duranta repens*, green tea, seaweeds, yeast

## Abstract

**Introduction:**

Fertilization is essential for the cultivation of ornamental and medicinal shrubs, but excessive application of chemical fertilizers contributes to environmental pollution and may adversely affect human health and beneficial soil microorganisms. Natural biostimulants such as active dry yeast (ADY), seaweed (SW), and green tea (GT) contain nutrients, bioactive compounds, and growth-promoting substances that can enhance plant growth. However, their combined use with reduced NPK fertilization has not previously been investigated in *Duranta repens* L. This study evaluated the potential of these natural biostimulants to reduce mineral fertilizer requirements while maintaining plant growth and quality.

**Methods:**

A randomized complete split-plot design was employed with three NPK fertilizer levels (50%, 75%, and 100% of the recommended dose) as main plots. The recommended dose consisted of 18, 12, and 6 g plant^−1^ of ammonium sulfate, calcium superphosphate, and potassium sulfate, respectively. Subplot treatments included an untreated control, ADY + GT, ADY + SW, SW + GT, and ADY + SW + GT.

**Results:**

The 75% NPK treatment significantly increased plant height, branch number, leaf area, chlorophyll index, shoot and root fresh and dry weights, total carbohydrates, and leaf nitrogen and phosphorus contents during both seasons. It also enhanced peroxidase and polyphenol oxidase activities, total phenolic compounds, and antioxidant activity, while catalase activity showed a non-significant increase. In contrast, the 100% NPK treatment produced the highest stem diameter, relative water content, and leaf potassium content. All biostimulant treatments significantly improved plant growth and biochemical characteristics compared with the untreated control. The interaction between fertilizer levels and biostimulants demonstrated that 75% NPK combined with ADY + SW + GT produced the highest shoot biomass, chlorophyll index, leaf nutrient contents, carbohydrates, total phenolics, antioxidant activity, and catalase activity. Meanwhile, 75% NPK with ADY + SW maximized plant height, branch number, and root biomass, whereas 75% NPK with SW + GT was the most effective in improving relative water content and oxidative enzyme activities.

**Discussion:**

These results demonstrate that integrating ADY, SW, and GT with 75% of the recommended NPK dose can reduce chemical fertilizer use while sustaining or enhancing the growth and physiological performance of *D. repens*.

## 1 Introduction

Ornamental plants are widely used for decorative purposes in gardens and various landscape design projects. These plants provide numerous beneficial impacts on the environment, including reducing CO_2_ levels and thermal emissions, along with remediating air pollutants, whether particulate matter or chemical contaminants. In addition, they represent important elements in tourist cities for several applications, such as public gardens, golf landscapes, and interior design.

*Duranta repens* L. (Fam. Verbenaceae) is an ornamental evergreen shrub native to open and clean forests in tropical and subtropical countries and in some temperate regions (Ganapaty et al., 1997; Hiradate et al., 1999). *D. repens* is commonly known as golden dewdrop or skyflower. It is an upright shrub or, occasionally, a small tree (3–4.5 m in height) (Little et al., 1974; Liogier, 1995). It bears axillary, pendent panicles (10–15 cm long) of small blue, lilac-blue, purple, or white flowers, mainly in summer. It grows outdoors in full sun and in moist, well-drained, moderately fertile soil, with moderate water requirements. It is used as an edging plant, for summer color, and as an interesting specimen. Its leaves, fruits, and flowers possess attractive aesthetic characteristics; therefore, it is widely used as an ornamental shrub in Egypt. The species also has economic importance as a source of timber, essential oils, tea, herbal medicine, tannins, and ornamental products (Nasir and Ray Ali, 1974). D. repens contains several important phytochemicals, including phenolics, steroids, flavonoids, alkaloids, glycosides, terpenoids, tannins, and saponins, which contribute to its therapeutic properties. The bioactive phytocompounds of *D. repens* exhibit valuable antioxidant, insecticidal, antimicrobial, and antifungal activities (Puri, 2018).

Nutritional and fertilization programs are among the major factors affecting the aesthetic characteristics of ornamental plants. The physicochemical properties and productivity of soil can be directly or indirectly influenced by fertilizer application (Li et al., 2020; Ren et al., 2020; Hou et al., 2023). Soil nutrient availability and citrus yield are strongly affected by fertilization practices; however, both nutrient availability and yield may initially increase and subsequently decline with excessive fertilizer application (Li et al., 2019). Nitrogen (N), phosphorus (P), and potassium (K) fertilizers are among the most commonly applied fertilizers. These nutrients play essential roles in plant cell metabolism, enzyme activity, and various chemical, biochemical, and physiological processes (Hawkesford et al., 2012). Moreover, fertilization significantly affects leaf functional traits (Bassi et al., 2018), including chlorophyll pigment content (Xiong et al., 2015), non-structural carbohydrates (Sun et al., 2020), and leaf nutrient composition (Liu et al., 2021). Zhang et al. (2023) reported that proper fertilization can significantly improve the yield and quality of blueberry fruits. Furthermore, several studies have demonstrated that moderate applications of N, P, and K fertilizers can positively enhance nutrient levels in plant leaves and soil, thereby increasing crop yield and quality (Zhang et al., 2020; Wan et al., 2021; Zhang et al., 2022). In addition, Jawad and Khalil (2024) reported that the application of 5 g NPK per rose transplant significantly increased leaf dry weight, chlorophyll content, flower diameter, and floral stem diameter in both spring and autumn.

Moreover, the excessive use of fertilizers may lead to soil acidification and severe nutrient imbalance, resulting in nutrient disorders in leaves and ultimately affecting crop yield and economic value (Raliya et al., 2017; Li et al., 2019; Xue et al., 2020). Furthermore, Yang et al. (2020) demonstrated that appropriate proportions of N, P, and K fertilizers can significantly enhance plant growth while reducing excessive fertilizer application. These findings are consistent with those reported by Mete et al. (2015) and Zhang et al. (2020). Similarly, excessive N fertilization negatively affects the physiological characteristics of *Musa nana* leaves and reduces soil nutrient content (Sun et al., 2020). Ultimately, both insufficient and excessive fertilization can adversely affect the physicochemical properties of soil, leaf characteristics, and plant productivity (Yang et al., 2020; Zhang et al., 2020). The highest significant plant height of *Bougainvillea* Dwarf Pink resulted from 0.5 g L^-1^ NPK + 1 g L^-1^ SW extract, while 0.5 g L^-1^ NPK + 0.5 g L^-1^ SW produced higher leaf fresh weight, bract head number, total bract number, and carotene content in bracts (Abdulrazzaq and Abdulmajeed, 2025). On the other hand, variations in optimal ratios of N, P, and K fertilizers have been observed within the same plant species under different environmental conditions, including soil type and fertility (Liu et al., 2024). The harmful effects associated with the excessive use of conventional fertilizers may be minimized through the application of natural extracts in combination with chemical N, P, and K fertilizers. Recently, the demand for biostimulant fertilizers has increased because they are safe for humans and livestock and are economically viable (Hussain et al., 2021; Sellami et al., 2025).

Active dry yeast (ADY), seaweed (SW), and green tea (GT) extracts have shown beneficial effects on plant growth and quality. *Saccharomyces cerevisiae* (ADY) is classified as a biostimulant fertilizer (Hakobyan et al., 2012; Arastech Far et al., 2013). Yeast is an important source of B vitamins and cytokinins (Matter and Abou-Sreea, 2016), in addition to carbohydrates, lipids, proteins, mineral nutrients, amino acids, and nucleic acids. It also contains pyridoxine, folic acid, vitamin B12, thiamine, and riboflavin (Nagodowithana, 1991; Abdelaal et al., 2017). The application of ADY extract alone or in combination with chitosan has been reported to enhance antioxidant enzyme activities and proline accumulation, thereby reducing oxidative stress caused by water deficiency in garlic plants (Abdelaal et al., 2021). Similarly, foliar spraying with yeast extract can increase antioxidant enzyme activities and reduce lipid peroxidation (Alzandi and Naguib, 2022). In addition, ADY extract promotes the production of secondary metabolites (Esmaeilzadeh Bahabadi and Sharifi, 2013), reduces reactive oxygen species (ROS), mitigates drought stress (Gholami et al., 2023) and salinity stress (El-Shawa et al., 2020), enhances cell division and elongation, and stimulates protein and nucleic acid synthesis, while also improving nutrient uptake (Nagodowithana, 1991; Matter and Abou-Sreea, 2016).

Previous studies have demonstrated that the use of yeast extract can improve growth characteristics, yield, and the chemical and biochemical composition of several plant species, including garlic (Shalaby and El-Ramady, 2014; Ali, 2017), calendula (El-Shawa et al., 2020), and Chinese carnation (Khudair and Hajam, 2021). Yeast extract at 3, 6, and 12 g L^-1^ enhanced plant height, leaf number, stem diameter, fresh weight, and K% and P% in *Eustoma russellianum* compared with untreated plants (Gabriel-Zunun et al., 2026). SW extracts are increasingly recognized as a promising novel class of agricultural inputs in the horticultural sector. SW extracts can be applied directly to the soil or used as compost amendments (Chojnacka et al., 2018), and they may also be administered as foliar sprays (Du Jardin, 2015). Recently, SW extracts have been identified as biostimulants that enhance nutrient absorption, improve resistance to abiotic stress, and increase crop quality parameters, regardless of their relative nutrient content. SW extracts are prepared from the extraction of numerous macroalgal species, and the extraction methodology determines the composition of the resulting complex mixtures of biologically active compounds (El Boukhari et al., 2020). These extracts contain various chemical and biochemical constituents, including proteins, vitamins, minerals, oils, fats, organic acids, hormones, antioxidants, pigments, and polysaccharides (Khan et al., 2008; Craigie, 2011; Michalak and Chojnacka, 2014). SW extracts also exert beneficial effects on soil structure, water retention, soil remediation, and soil microflora, while serving as important sources of nutrients and plant growth regulators (Du Jardin, 2015). Consequently, the application of SW has demonstrated positive effects on plant growth, development, and quality characteristics, as reported in studies on rose plants (Kularathne et al., 2021), *Lantana camara* and *Abelia × grandiflora* (Loconsole et al., 2022), *Citrullus lanatus* (Radwan et al., 2023), kiwifruit (Rana et al., 2023), and strawberry (Bagh et al., 2024). Awad et al. (2025) found that 2 mL^-1^ SW or 2 g L^-1^ ADY improved vegetative growth and yield components of okra plants. Similarly, 20 mL^-1^ SW extract strongly enhanced plant height, leaf area, nut dry weight, nuts per plant, and seed number in roselle (cultivar eggplant) (Rumi and Jarallah, 2025).

The use of plants as organic fertilizers is considered an important agricultural approach for improving the nutritional status of plants, particularly under unfavorable environmental conditions that may affect plants during different stages of their life cycle. Consequently, increasing attention has been directed toward the use of plant-based organic fertilizers and their application as foliar sprays to facilitate absorption and translocation within different plant tissues (Niu et al., 2021; Vasundhara and Chhabra, 2021).

GT, *Camellia sinensis* L., is a plant-derived organic fertilizer. GT is well known for its antioxidant properties. Bachrach and Wang (2002) reported that GT contains polyphenolic compounds accounting for approximately 30% of the leaf dry weight, with flavanols representing the major group of polyphenols, among which (−)-epigallocatechin-3-gallate is the most abundant. Furthermore, Armstrong et al. (2020) demonstrated that the beneficial effects of GT extract are attributed to its high content of readily absorbable nutrients, vitamins, and antioxidant polyphenolic compounds, in addition to its role in stimulating enzymatic activities that reduce oxidative stress. Moreover, caffeine, tannins, and essential oils are important constituents of GT extract. The major active polyphenols in GT extract include catechin, epigallocatechin, and epigallocatechin gallate (Nagle et al., 2006; Asadi et al., 2013). Numerous studies have reported the positive effects of GT extract on plant growth and on the chemical and biochemical composition of plants, including *Allium cepa* (Çavuşoğlu, 2020), Aralia plants (Moustafa, 2020), Barhee date palm (Abdel Aal et al., 2021), and *Ricinus communis* (Hassan and Ibrahim, 2024).

Further research is required to achieve a comprehensive understanding of the underlying mechanisms and to determine the optimal combination of ADY, SW, and GT extracts with conventional mineral fertilizers. Recently, limited information has been available regarding fertilization programs for ornamental shrubs. Therefore, the present study focused on evaluating the growth performance and chemical and biochemical composition of *Duranta repens* in response to different NPK fertilizer doses, natural extracts (ADY, SW, and GT), and their interactions. The study aimed to identify the most effective fertilization treatment for improving the aesthetic characteristics of *D. repens* for landscape and medicinal uses, while reducing the overuse of chemical fertilizers through the application of natural biostimulants, including ADY, SW, and GT, to minimize environmental pollution of the air, soil, and groundwater.

## 2 Materials and methods

Throughout the two successive seasons of 2023 and 2024, pot experiments were conducted at the Farm of the Horticulture Department of the Faculty of Agriculture, Kafrelsheikh University, Egypt. The study was conducted to evaluate the performance of vegetative growth, enzyme activities, total phenolic compounds, antioxidant activity (AOA), relative water content (RWC), and chemical composition of *Duranta repens* L. in response to NPK doses, natural extracts (ADY, SW, and GT), and their interactions. The coordinates of the farm location are 31°61´ N, 30°57´ E, at an elevation of 6 m above sea level. According to the nearby Sakha meteorological station, the meteorological data for the experimental seasons of 2023 and 2024 were as follows: total rainfall (111.68 and 103.28 mm), average relative humidity (57.7% and 59.6%), average maximum temperature (29.6 °C and 30.4 °C), and average minimum temperature (15.0 °C and 14.3 °C), respectively.

### 2.1 Plant material and planting date

*Duranta repens* transplants were 3 months old, measured 19 ± 1 cm in height, and were obtained from the nursery of the Horticulture Department, Faculty of Agriculture, Kafrelsheikh University, for use in this research. The transplants were planted in plastic pots (one transplant/pot) with a diameter and height of 40 and 32 cm, respectively. The pots were filled with 9 kg/pot of a clay and sand mixture (1:1, v/v) on 1 April in the 2023 and 2024 seasons. The plants were placed under open-field conditions and irrigated with tap water (pH 7.2 and Electricity Conductivity (EC) 0.37 ds/m) every 3 days to reach field capacity. Weed and insect control measures were applied during the experimental seasons.

### 2.2 Medium analysis

Before planting, the growth medium was analyzed for physical and chemical parameters. The medium was air-dried, homogenized, and carefully ground using a mortar and pestle. Fractions smaller than 2 mm were obtained after the medium sample was sieved through a stainless-steel mesh (Cools and De Vos, 2011). The particle-size distribution of the medium was determined by the hydrometer method (US21CFR1040.10AND1040.11, USA) (Gee and Bauder, 1986). The medium pH was measured in a suspension (1:2.5; medium: distilled water) after 30 min using a pH meter (JENEWY3510, Staffordshire, UK) according to Black et al. (1965). To estimate EC, organic matter (OM), and available N, P, and K, a 1:5 ratio was obtained by mixing 20 g of air-dried medium with 100 mL of distilled water. The suspension was allowed to stand for 24 h before being filtered.

The medium EC was estimated using an EC meter (MI170, SZ, Egged, Hungary, Italy) (Jackson, 1973). Medium OM was evaluated according to the method of Nelson and Sommers (1996). The available N content was determined by the Micro–Kjeldahl procedure (DNB.1500NPS.N.33848, RAYPA, Spain) (Bremner and Mulvaney, 1983). Available P was measured by the procedure of Olsen and Sommers (1982). Available K was evaluated using a PSC7 flame photometer (JENEWY, Staffordshire, UK) (Black et al., 1965).

The growth medium contained 40.05% clay, 37.03% sand, and 22.92% silt, with a clayey sand texture. It had a pH of 7.84, EC of 1.53 mmhos/ds, organic matter of 1.33%, and available N, P, and K contents of 1.56, 2.27, and 163 mg/kg, respectively.

### 2.3 Experimental layout

A randomized complete split-plot design with three replicates was used to conduct the experiments (Snedecor and Cochran, 1989). A total of 15 treatment combinations (3 NPK doses x 5 natural extract levels) were included in each plot (replicate). Two plants were used for each treatment within each plot (replicate). Thus, there were six plants for the three replicates (plots). The experiment included a total of 90 plants in each season.

### 2.4 Fertilizers applied

#### 2.4.1 Mineral fertilizers

Ammonium sulfate (20.5% N), calcium superphosphate (15.5% P_2_O_5_), and K sulfate (48% K_2_O) were utilized as sources of N, P, and K at rates of 18, 12, and 6 g/pot, respectively, as the recommended 100% NPK dose. Calcium superphosphate was added as a single dose during medium preparation before planting. The N and K fertilizer doses were divided into six equal rates and applied monthly from 1 May to 1 October in both seasons. These fertilizers were added to the medium by drenching.

#### 2.4.2 Natural extracts

ADY at 3 g L^-1^, SW (algae of *Spirulina platensis* and *Ecklonia maxima*, obtained from the Laboratory of Horticultural Botany, Department of Horticulture, Faculty of Agriculture, Kafrelsheikh University) at 1.5 mL L^-1^, and GT (*Camellia sinensis*) at 0.5 g L^-1^ were used as natural extracts. The extracts were applied as foliar sprays three times at one-month intervals, beginning on 2 May during each experimental season. A wetting agent (Triton B) was added at 0.05 L^-1^ to all extracts during application. Spraying was conducted in the morning, and the plants were sprayed until runoff. The concentrations of ADY, SW, and GT were based on the previous studies of Mohamed et al. (2021), Rana et al. (2023), and Abdel Aal et al. (2021), respectively.

##### 2.4.2.1 Natural extract preparation

The ADY extract was prepared by inoculating 3 g of yeast in 1 L of nutrient broth and incubating it for 48 h in the dark. **Table 1** shows the composition of ADY.

**Table 1:** Composition of ADY used.

Minerals %				Total protein (%)	Total carbohydrates (%)	Hormones (μg/mL)	
N	1.02	Zn	0.04	5.2	4.8	IAA	GA_s_
P	0.09	Cu	0.03			0.7	0.3
K	0.40	Fe	0.12				
Ca	0.03	Mn	0.05				
Na	0.008	Mo	0.0001				
Mg	0.01	B	0.013				

The SW extract was prepared by drying the seaweeds and transforming them into powder, followed by thorough grinding using a laboratory blender (Waring Commercial, USA). SW powder was mixed with distilled water at a ratio of 1: 20 (w/v), then autoclaved (Gemmy, Taiwan) at 121 °C and 15 Ibs/Sq for 20 min. After that, the warm suspension was filtered through double-layered cheesecloth and left to cool to 4 °C. A centrifuge (Legend Micro-17/17R, Thermo Electron Corporation, Germany) was used to centrifuge the filtrate at 5,000 x g for 15 min. The supernatant was considered a 100% SW liquid extract (Sutharsan et al., 2014). The composition of the SW extract is presented in **Table 2**.

**Table 2:** Seaweed extract composition.

Macroelements (%)		Microelements (mg/L)		Hormones mg/L		Lactic acid (%)	Amino acids (%)	Matinol (%)
N	1	Fe	2,000	Cytokinines	10	5	2.2	10
P	5	Mn	1,000	IAA	200			
K	10	Zn	3,000	Gibberellins	0.04			

The GT extract was prepared by soaking dried GT leaves (which were bought from a commercial store that imported them from China) (20 g) in distilled water (200 mL) in a 500-mL flask at 25 °C for 1 h. The mixture was then shaken at medium speed for 2 h and left to settle for 1 h. Whatman No. 4 filter paper was used to filter the mixture to separate the residue from the filtrate.

Afterward, 5 mL of the filtrate was diluted to one liter with distilled water to prepare the working concentration (0.5 g L^-1^) (Harborne, 1954). The extract was kept in a dark bottle and stored at 4 °C until use.

### 2.5 Experimental treatments applied

#### 2.5.1 NPK doses

NPK doses of 50%, 75%, and 100% of the recommended doses mentioned above were assigned to the main plots.

#### 2.5.2 Biostimulant extract treatments

The subplot treatments were as follows: control (deionized water); 3 g L^-1^ ADY + 0.5 g L^-1^ GT; 3 g L^-1^ ADY + 1.5 ml L^-1^ SW; 1.5 ml L^-1^ SW + 0.5 GT; and 3 g L^-1^ ADY + 1.5 ml L^-1^ SW + 0.5 g L^-1^ GT.

### 2.6 Data recorded

The following data were measured on 1 November of each season.

#### 2.6.1 Vegetative parameters

Plant height (PH) (cm) was measured from soil surface to plant apex; branch number/plant and primary stem diameter (PSD) (cm) were measured at 3 cm above the soil surface. Leaf area (cm^2^) was measured using a CI-202 laser area meter (CIDBIO-Science, Cams WA). Chlorophyll index (CI) (SPAD units) was estimated using a portable leaf chlorophyll meter (SPAD-501: Minolta Crop, Osaka, Japan) (Markwell et al., 1995). Shoots (leaves + stems) and root fresh and dry weights were also determined. The plant parts were separately washed twice with tap water to remove dust and soil, then rinsed by distilled water. The plant parts were dried in an oven for 24 h at 80 °C to determine the dry weight (Rautio et al., 2010).

#### 2.6.2 Leaf relative water content percentage (RWC%)

Five discs of fresh *D. repens* samples (1 cm diameter per disc) were weighed. The discs were then floated in distilled water for 4 h to become fully turgid, and the turgid weight was determined. The dry weight of the discs was determined after drying at 70 °C for 24 h (Barrs, 1968).

Leaf RWC% was calculated as follows:


$$\text{Leaf}\text{ RWC}\%=\frac{Freshweight-Dryweight}{Turgidweight-Dryweight}\times 100$$

#### 2.6.3 Enzymes activity assay

The homogenized fresh *D. repens* leaves (0.5 g) were centrifuged at 12,000 × g for 20 min at 4 °C. After that, catalase (CAT), peroxidase (POD), and polyphenol oxidase (PPO) activities were estimated in the obtained supernatant. CAT was measured at 240 nm as μmol min^-1^ mg protein^-1^ based on the rate of H_2_O_2_ consumption (Havir and McHale, 1987). POD activity was estimated according to the procedure of Castillo et al. (1984) and expressed as μmol min^-1^ mg protein^-1^. The reaction mixture contained 10 mM phosphate buffer (pH 6.1), 12 mM H_2_O_2_, 96 mM guaiacol and the enzyme extract. A complete reaction mixture without H_2_O_2_ was used as a blank. After 1 min, the absorbance was recorded at 470 nm, and the activity was calculated using an extinction coefficient of 26.6 mM^-1^ cm^-1^. PPO activity was evaluated according to the procedure of Malik and Singh (1980). The reaction mixture included 3.0 mL of 0.01 M buffer at pH 6.0 and 100 μL of crude enzyme extract. Changes in absorbance were evaluated at 495 nm every 30 s over a 3-min period. PPO activity was determined as the increase in absorbance min^-1^ g^-1^ fresh weight. Enzymatic activities were determined in the 2024 season only.

#### 2.6.4 Total phenolic compounds and antioxidant activity

The methanol extract of air-dried, ground leaves was prepared to estimate total phenolic compounds (TPC_S_). The content of TPC_S_ was estimated in the crude extract using an external calibration curve based on gallic acid with the Folin-Ciocalten reagent (Singleton et al., 1999). Then, the Folin-Ciocalteu reagent was added to 0.2 mL of each extract. After a few minutes, 1 mL of 15% sodium carbonate was added, and the mixture was left to stand at room temperature for 4 h. After that, the absorbance was recorded at 760 nm using a spectrophotometer (Thermo Fisher Scientific, Waltham, MA, USA; model 4001/4). The TPC_S_ was determined as mg of gallic acid equivalents per gram of dry weight (mg GAE/g DW). Each result represented the average of five independent measurements per fraction (Martínez-Esplá et al., 2014).

The extracts of the dried leaves were used to assay antioxidant activity (AOA) using a modified version of the 2,2-diphenyl-1-picrylhydrazyl (DPPH) radical-scavenging assay, according to the method of Binsan et al. (2008). A protein solution (0.1%) in 5 mM phosphate-buffered saline (PBS, pH 7.2) was added to an equal volume (1:1, v/v) of 0.15 mM DPPH dissolved in ethanol (95%). The mixture was carefully vortexed and incubated in the dark at room temperature for 30 min. The resulting absorbance was recorded at 570 nm using a spectrophotometer (Helios Gamma; Thermo Fisher Scientific). A blank was prepared in the same manner, except that PBS was used instead of the sample. Trolox was used to generate the calibration curve, with levels ranging from 12.5–100 μM. AOA was evaluated as Trolox equivalents (TE)/mg of dry leaves. TPC_S_ and AOA were determined in the 2024 season only.

#### 2.6.5 Leaf chemical determinations

The percentages of N, P, and K and total carbohydrates (TCs) were estimated in the dried leaves after drying in an oven at 80 °C for 24 h. A metal-free mill (IK-a- Werke, M 20, Germany) was used to grind the dried leaves to obtain a uniform powder. Then, 5 mL of sulfuric acid (95%) was gently added to 0.2 g of sample, and the mixture was heated for 10 min on a sand hotplate. After that, to obtain a clear solution, 0.5 mL of perchloric acid was added dropwise while heating was continued. The solution was left to cool, then it was filtered and diluted to 50 mL with deionized water (Evenhuis and Dewaard, 1980). Nitrogen percentage was estimated according to the micro-Kjeldahl method (Chemists and Horwitz, 1990). P% was quantified using a spectrophotometer (GT 80^+^, UK) (Murphy and Riley, 1962). K% was determined using an atomic absorption spectrophotometer (Cottenie et al., 1982). The procedure of Herbert et al. (1971) was applied to measure the percentage of TCs.

### 2.7 Statistical analysis

The results were subjected to analysis of variance (ANOVA), and SAS software (version 6.12; SAS Institute Inc, Cary, NC, USA) was used for data analysis. Mean separation (± standard error) was performed. using a split-plot design model incorporating NPK doses, natural biostimulant treatments, and their interaction. Significant differences among means were evaluated (F- value) at *P* ≤ 0.05 by Duncan’s multiple range test (DMRT).

## 3 Results

### 3.1 Vegetative parameters

Data in **Tables 3**–**5** show the effects of NPK doses, natural extracts, and their interactions on plant height (PH), number of branches per plant (NB), primary stem diameter (PSD), area per leaf (AL), chlorophyl index (CI), and fresh and dry weights of shoots and roots (FWS, DWS, FWR, and DWR). Notably, the 75% NPK dose resulted in the highest significant values of PH (51.13 and 53.30 cm), NB (11.93 and 12.80 branches/plant), AL (46.14 and 47.73 cm^2^), CI (17.67 and 18.22 SPAD units), FWS (95.76 and 96.20 g/plant), DWS (24.00 and 24.70 g/plant), FWR (40.01 and 41.05 g/plant), and DWR (12.41 and 12.70 g/plant) (*P* ≤ 0.05) in both seasons, respectively. The highest PSD values resulted from using the 100% NPK dose (0.62 and 0.64 cm). The differences between the 100% and 75% NPK doses did not reach statistical significance (*P* ≤ 0.05) for NB and DWS in the first season, AL in the second season, and FWS in both seasons. In contrast, the plants that received the 50% NPK dose had the lowest significant values for these parameters in both seasons.

**Table 3:** Plant height, number of branches per plant, and primary stem diameter of *D. repens* as affected by NPK doses, natural extracts, and their interaction in the 2023 and 2024 seasons.

NPK doses (g/plant)	Natural extracts	Plant height (cm)		Number branches/plant		Primary stem diameter (cm)	
		1^st^ season	2^nd^ season	1^st^ season	2^nd^ season	1^st^ season	2^nd^ season
50% NPK		46.40 ± 0.76 C	48.30 ± 0.29 C	7.81 ± 0.23 B	8.50 ± 0.29 C	0.48 ± 0.020 C	0.49 ± 0.010 C
75% NPK		51.13 ± 1.60 A	53.30 ± 0.75 A	11.93 ± 0.35 A	12.80 ± 0.23 A	0.54 ± 0.006 B	0.56 ± 0.006 B
100% NPK		48.67 ± 0.74 B	51.90 ± 1.10 B	11.93 ± 0.18 A	12.60 ± 0.58 B	0.62 ± 0.005 A	0.64 ± 0.004 A
	Control	41.77 ± 1.64 E	43.16 ± 0.29 E	6.67 ± 0.96 E	7.50 ± 0.48 E	0.42 ± 0.011 E	0.43 ± 0.007 E
	3g L^-1^ADY+0.5g L^-1^GT	46.44 ± 1.87 D	49.00 ± 0.58 D	11.34 ± 1.39 B	12.66 ± 0.38 B	0.52 ± 0.022 D	0.53 ± 0.016 D
	3g L^-1^ADY+1.5mlL^-1^SW	53.67 ± 3.18 A	57.00 ± 1.73 A	13.11 ± 0.48 A	13.33 ± 0.37 A	0.56 ± 0.012 C	0.58 ± 0.019 C
	1.5mlL^-1^SW +0.5g L^-1^GT	49.33 ± 0.51 C	52.16 ± 0.67 C	11.33 ± 0.58 C	12.16 ± 0.67 C	0.61 ± 0.015 B	0.62 ± 0.010 B
	3g L^-1^ADY +1.5mlL^-1^SW +0.5g L^-1^GT	52.44 ± 2.38 B	54.50 ± 1.44 B	10.22 ± 0.87 D	10.83 ± 0.10 D	0.63 ± 0.012 A	0.66 ± 0.013 A
		**	**	**	**	**	**
50% NPK	Control	40.00 ± 3.06 n	41.00 ± 2.31 n	6.00 ± 0.58 l	6.50 ± 0.28 k	0.33 ± 0.009 n	0.33 ± 0.003 k
	3g L^-1^ADY+0.5g L^-1^GT	44.33 ± 2.85 k	46.00 ± 2.31 k	7.67 ± 1.44 i	9.00 ± 0.57 g	0.45 ± 0.032 l	0.46 ± 0.026 i
	3g L^-1^ADY+1.5mlL^-1^SW	50.33 ± 5.49 f	55.00 ± 2.89 e	8.00 ± 0.58 h	8.50 ± 0.29 h	0.48 ± 0.035j	0.51 ± 0.003 g
	1.5mlL^-1^SW +0.5g L^-1^GT	47.33 ± 1.45 i	48.50 ± 0.87 i	9.67 ± 0.88 g	10.50 ± 0.28 f	0.54 ± 0.025 i	0.55 ± 0.020 f
75% NPK	3g L^-1^ADY +1.5mlL^-1^SW +0.5g L^-1^GT	50.00 ± 1.15 g	51.00 ± 0.58 h	7.33 ± 0.67 j	8.00 ± 0.00 i	0.58 ± 0.035 g	0.61 ± 0.003 d
	Control	42.33 ± 1.45 m	43.50 ± 0.87 m	6.67 ± 0.88 k	7.50 ± 0.28 j	0.39 ± 0.009 m	0.40 ± 0.006 j
	3g L^-1^ADY+0.5g L^-1^GT	50.00 ± 4.04 a	53.50 ± 2.02 g	12.67 ± 1.20 d	13.50 ± 0.88 c	0.46 ± 0.009 k	0.47 ± 0.003 h
	3g L^-1^ADY+1.5mlL^-1^SW	57.67 ± 1.20 a	58.50 ± 0.87 a	17.67 ± 0.67 a	18.00 ± 0.57 a	0.55 ± 0.032 h	0.56 ± 0.026 e
	1.5mlL^-1^SW +0.5g L^-1^GT	49.67 ± 0.67 h	54.00 ± 0.58 f	11.33 ± 1.45 f	12.50 ± 0.87 d	0.62 ± 0.024 f	0.64 ± 0.017 c
100% NPK	3g L^-1^ADY +1.5mlL^-1^SW +0.5g L^-1^GT	56.00 ± 4.62 b	57.00 ± 2.31 c	11.33 ± 1.86 f	12.50 ± 1.44 d	0.69 ± 0.042 a	0.73 ± 0.012 a
	Control	43.00 ± 3.06 l	45.00 ± 2.31 l	7.33 ± 0.58 j	8.50 ± 0.87 h	0.54 ± 0.033 i	0.56 ± 0.029 e
	3g L^-1^ADY+0.5g L^-1^GT	45.00 ± 3.61 j	47.50 ± 2.60 j	13.67 ± 1.86 b	15.50 ± 0.9 b	0.65 ± 0.032 c	0.66 ± 0.026 b
	3g L^-1^ADY+1.5mlL^-1^SW	53.00 ± 4.73 c	57.50 ± 1.44 b	13.67 ± 1.20 b	13.50 ± 0.87 c	0.64 ± 0.033 d	0.66 ± 0.029 b
	1.5mlL^-1^SW +0.5g L^-1^GT	51.00 ± 3.06 e	54.00 ± 0.58 f	13.00 ± 1.00 c	13.50 ± 0.86 c	0.66 ± 0.033 b	0.66 ± 0.032 b
	3g L^-1^ADY +1.5mlL^-1^SW +0.5g L^-1^GT	51.33 ± 1.86 d	55.50 ± 1.44 d	12.00 ± 1.15 e	12.00 ± 1.16 e	0.63 ± 0.042 e	0.66 ± 0.029 b

Means in a column with similar letters are not significantly different (*P* ≤ 0.05) according to DMRT.

ADY, Active dry yeast; SW, seaweed; GT, green tea.

Highly significant = **; significant = *; non-significant = ns.

**Table 4:** Leaf area and shoot fresh and dry weights of *D. repens* as affected by NPK doses, natural extracts, and their interaction in the 2023 and 2024 seasons.

NPK doses (g/plant)	Natural extracts	Area/leaf (cm2)		Shoots FW/plant (g)		Shoots DW/plant (g)	
		1^st^ season	2^nd^ season	1^st^ season	2^nd^ season	1^st^ season	2^nd^ season
50% NPK		33.12 ± 0.35 C	34.66 ± 0.51 B	66.20 ± 3.30 B	70.95 ± 2.08 B	16.68 ± 0.98 B	17.95 ± 0.20 C
75% NPK		46.14 ± 0.85 A	47.73 ± 0.60 A	95.76 ± 1.69 A	96.20 ± 0.32 A	24.00 ± 0.18 A	24.70 ± 0.14 A
100% NPK		45.59 ± 0.19 B	46.77 ± 0.01 A	93.16 ± 1.18 A	93.43 ± 0.58 A	23.54 ± 0.29 A	23.88 ± 0.01 B
	Control	32.17 ± 0.69 E	34.00 ± 1.01 E	54.72 ± 0.34 D	55.41 ± 0.53 C	13.82 ± 0.22 D	14.01 ± 0.08 D
	3g L^-1^ADY+0.5g L^-1^GT	39.55 ± 1.89 D	41.48 ± 0.33 D	81.11 ± 1.87 C	85.66 ± 0.77 B	20.30 ± 0.57 C	21.52 ± 0.14 C
	3g L^-1^ADY+1.5mlL^-1^SW	41.57 ± 0.45 C	42.77 ± 0.60 C	92.83 ± 1.36 B	94.91 ± 0.14 A	23.29 ± 0.36 B	23.96 ± 0.02 B
	1.5mlL^-1^SW +0.5g L^-1^GT	45.77 ± 0.80 B	46.58 ± 0.64 B	97.83 ± 1.36 A	99.08 ± 2.07 A	24.58 ± 0.62 A	25.31 ± 0.26 A
	3g L^-1^ADY +1.5mlL^-1^SW +0.5g L^-1^GT	49.07 ± 0.64 A	50.43 ± 0.07 A	98.72 ± 2.55 A	99.22 ± 1.73 A	25.04 ± 0.53 A	26.08 ± 0.24 A
50% NPK		**	**	**	**	**	**
	Control	28.21 ± 2.40 o	30.32 ± 1.15 o	49.50 ± 0.87 h	50.25 ± 0.43 h	12.43 ± 0.26 h	12.65 ± 0.05 j
	3g L^-1^ADY+0.5g L^-1^GT	30.41 ± 1.30 m	31.21 ± 1.03 m	49.50 ± 5.48 h	64.25 ± 2.74 g	12.23 ± 1.24 h	16.37 ± 0.45 h
	3g L^-1^ADY+1.5mlL^-1^SW	29.35 ± 1.21 n	30.52 ± 0.30 n	71.00 ± 3.46 f	74.25 ± 1.73 f	17.95 ± 1.04 f	18.47 ± 0.46 g
	1.5mlL^-1^SW +0.5g L^-1^GT	34.18 ± 2.97 k	36.13 ± 2.23 k	86.00 ± 8.08 e	90.75 ± 1.87 e	21.93 ± 2.41 e	23.05 ± 0.24 f
75% NPK	3g L^-1^ADY +1.5mlL^-1^SW +0.5g L^-1^GT	43.43 ± 1.72 h	45.15 ± 0.09 i	75.00 ± 0.58 f	75.50 ± 0.29 f	18.88 ± 0.07 f	19.21 ± 0.27 g
	Control	32.47 ± 1.97 l	34.21 ± 0.92 l	55.66 ± 1.20 gh	56.50 ± 0.87 gh	13.95 ± 0.29 gh	14.46 ± 0.18 i
	3g L^-1^ADY+0.5g L^-1^GT	41.33 ± 4.06 i	45.24 ± 1.06 h	100.50 ± 0.87 cd	98.25 ± 0.43 cde	25.01 ± 0.12 cd	24.40 ± 0.25 ef
	3g L^-1^ADY+1.5mlL^-1^SW	47.39 ± 2.10 f	49.03 ± 1.31 d	105.83 ± 3.90 abc	108.25 ± 1.88 ab	26.29 ± 0.70 abc	27.88 ± 0.53 cd
	1.5mlL^-1^SW +0.5g L^-1^GT	54.37 ± 0.64 b	54.92 ± 0.32 b	105.50 ± 3.75 abc	104.00 ± 4.04 bc	26.29 ± 0.61 abc	26.58 ± 0.74 d
100% NPK	3g L^-1^ADY +1.5mlL^-1^SW +0.5g L^-1^GT	55.15 ± 0.12 a	55.25 ± 0.06 a	111.33 ± 1.86 a	114.00 ± 3.46 a	28.47 ± 0.84 a	30.21 ± 0.18 a
	Control	35.82 ± 1.91 j	37.47 ± 0.96 j	59.00 ± 0.58 g	59.50 ± 0.29 g	15.08 ± 0.46 g	14.94 ± 0.04 i
	3g L^-1^ADY+0.5g L^-1^GT	46.90 ± 1.51 g	48.01 ± 1.03 g	93.33 ± 1.45 de	94.50 ± 0.87 de	23.67 ± 0.68 de	23.79 ± 0.37 f
	3g L^-1^ADY+1.5mlL^-1^SW	47.96 ± 0.84 e	48.78 ± 0.18 e	101.66 ± 0.88 c	102.50 ± 0.29 bcd	25.65 ± 0.08 bcd	25.54 ± 0.04 cd
	1.5mlL^-1^SW +0.5g L^-1^GT	48.65 ± 0.005 c	48.69 ± 0.02 f	102.00 ± 0.58 bc	102.50 ± 0.29 bcd	25.53 ± 0.15 bcd	26.29 ± 0.61 c
	3g L^-1^ADY +1.5mlL^-1^SW +0.5g L^-1^GT	48.63 ± 0.34 d	50.92 ± 0.17 c	109.83 ± 5.42 ab	108.16 ± 1.44 ab	27.79 ± 1.12 ab	28.85 ± 0.93 bc

Means with the same letters in a column are not significantly different (P ≤ 0.05) according to DMRT.

ADY, Active dry yeast; SW, seaweed; GT, green tea.

Highly significant = **; significant = *; non-significant = ns.

**Table 5:** Chlorophyll index and root fresh and dry weights of *D. repens* as affected by NPK doses, natural extracts, and their interaction in the 2023 and 2024 seasons.

NPK doses (g/plant)	Natural extracts	Chlorophyll index (SPAD units)		Roots FW/plant (g)		Roots DW/plant (g)	
		1^st^ season	2^nd^ season	1^st^ season	2^nd^ season	1^st^ season	2^nd^ season
50% NPK		14.49 ± 0.31 C	15.12 ± 0.05 C	31.80 ± 0.82 C	34.20 ± 0.40 C	10.11 ± 0.42 B	10.57 ± 0.20 C
75% NPK		17.67 ± 0.22 A	18.22 ± 0.13 A	40.01 ± 0.75 A	41.05 ± 0.61 A	12.41 ± 0.20 A	12.70 ± 0.12 A
100% NPK		15.98 ± 0.42 B	17.00 ± 0.10 B	36.27 ± 0.19 B	37.35 ± 0.72 B	11.69 ± 0.28 B	11.59 ± 0.13 B
	Control	11.84 ± 0.08 E	12.27 ± 0.24 E	26.11 ± 0.29 C	26.67 ± 0.38 D	08.06 ± 0.31 E	08.22 ± 0.13 E
	3g L^-1^ADY+0.5g L^-1^GT	18.44 ± 0.57 B	19.28 ± 0.49 B	35.50 ± 0.35 B	36.08 ± 0.14 C	10.99 ± 0.42 D	11.10 ± 0.24 D
	3g L^-1^ADY+1.5mlL^-1^SW	12.57 ± 0.22 D	13.11 ± 0.19 D	41.11 ± 1.44 A	42.33 ± 0.77 A	13.57 ± 0.58 A	13.10 ± 0.23 A
	1.5mlL^-1^SW +0.5g L^-1^GT	14.20 ± 0.14 C	14.77 ± 0.38 C	40.05 ± 1.03 A	41.17 ± 0.87 B	12.70 ± 0.23 B	12.92 ± 0.37 B
	3g L^-1^ADY +1.5mlL^-1^SW +0.5g L^-1^GT	22.98 ± 0.15 A	24.45 ± 0.61 A	37.50 ± 0.83 B	41.42 ± 0.72 AB	11.69 ± 0.14 C	12.76 ± 0.21 C
50% NPK		**	**	**	**	**	**
	Control	11.37 ± 0.38 n	11.65 ± 0.26 o	24.00 ± 0.88 g	24.50 ± 0.29 h	07.33 ± 0.31 o	07.55 ± 0.09 o
	3g L^-1^ADY+0.5g L^-1^GT	16.10 ± 1.32 f	17.35 ± 0.43 f	32.67 ± 1.20 def	33.50 ± 0.87 f	10.15 ± 0.71 k	10.32 ± 0.51 l
	3g L^-1^ADY+1.5mlL^-1^SW	12.53 ± 0.75 k	13.15 ± 0.42 k	36.33 ± 0.88 bcd	37.00 ± 0.58 de	11.97 ± 0.54 f	11.48 ± 0.25 f
	1.5mlL^-1^SW +0.5g L^-1^GT	14.43 ± 0.60 g	14.85 ± 0.43 h	34.33 ± 1.86 cde	35.50 ± 1.44 e	11.26 ± 0.24 i	11.00 ± 0.22 k
75% NPK	3g L^-1^ADY +1.5mlL^-1^SW +0.5g L^-1^GT	18.03 ± 1.08 e	18.60 ± 0.92 e	31.67 ± 0.88 ef	40.50 ± 0.29 c	09.83 ± 0.46 l	12.48 ± 0.26 f
	Control	12.50 ± 0.45 l	12.95 ± 0.03 l	24.33 ± 0.58 g	25.00 ± 0.58 h	07.52 ± 0.30 n	07.66 ± 0.13 n
	3g L^-1^ADY+0.5g L^-1^GT	18.93 ± 0.68 d	19.30 ± 0.58 d	37.66 ± 1.20 bc	38.00 ± 1.15 d	11.63 ± 0.27 g	11.70 ± 0.17 h
	3g L^-1^ADY+1.5mlL^-1^SW	12.97 ± 0.52 i	13.30 ± 0.40 j	47.00 ± 2.31 a	49.00 ± 1.14 a	14.77 ± 0.82 a	15.13 ± 0.22 a
	1.5mlL^-1^SW +0.5g L^-1^GT	13.73 ± 0.86 h	14.35 ± 0.61 i	47.00 ± 0.58 a	47.50 ± 0.29 a	14.43 ± 0.44 b	14.94 ± 0.61 b
100% NPK	3g L^-1^ADY +1.5mlL^-1^SW +0.5g L^-1^GT	30.20 ± 1.40 a	31.20 ± 0.98 a	44.50 ± 1.44 a	45.75 ± 0.72 b	13.68 ± 0.22 d	14.08 ± 0.41 c
	Control	11.67 ± 0.56 m	12.20 ± 0.17 n	30.00 ± 0.58 f	30.50 ± 0.29 g	09.33 ± 0.49 m	09.45 ± 0.23 m
	3g L^-1^ADY+0.5g L^-1^GT	20.30± 1.01 c	21.20 ± 0.46 c	36.17 ± 0.60 bcd	36.75 ± 0.14f de	11.18 ± 0.48 j	11.29 ± 0.23 j
	3g L^-1^ADY+1.5mlL^-1^SW	12.77± 0.18 j	12.90 ± 0.12 m	40.00 ± 1.15 b	41.00 ± 0.58 c	13.98 ± 0.67 c	12.68 ± 0.24 e
	1.5mlL^-1^SW +0.5g L^-1^GT	14.43 ± 0.76 g	15.15 ± 0.26 g	38.83 ± 1.88 b	40.50 ± 0.87 c	12.40 ± 0.20 e	12.82 ± 0.46 d
	3g L^-1^ADY +1.5mlL^-1^SW +0.5g L^-1^GT	20.73 ± 2.82 b	23.55 ± 0.09 b	36.33 ± 2.03 bcd	38.00 ± 1.15 d	11.57 ± 0.38 h	11.73 ± 0.48 g

Means with the same letters in a column are not significantly different (*P ≤ 0.05*) according to DMRT.

ADY, Active dry yeast; SW, seaweed; GT, green tea.

Highly significant = **; significant = *; non-significant = ns.

Similarly, all natural extracts used significantly increased the aforementioned vegetative traits relative to the respective control (**Tables 3**-**5**). Application of 3 g L^-1^ ADY + 1.5 ml L^-1^ SW significantly improved PH (53.67 and 57.00 cm), NB (13.11 and 13.33 branches/plants), FWR (41.11 and 42.33 g/plant), and DWR (13.57 and 13.10 g/plant). Furthermore, the highest significant values of PSD (0.63 and 0.66 cm), AL (49.07 and 50.43 cm^2^), CI (22.98 and 24.45 SPAD units), FWS (98.72 and 99.22 g/plant), and DWS (25.04 and 26.08 g/plant) were achieved with the application of 3 g L^-1^ ADY + 1.5 ml L^-1^ SW + 0.5 g L^-1^ GT in both seasons. The differences among the natural extract treatments were statistically significant (*P ≤ 0.05*) for the majority of vegetative traits.

The interaction between NPK doses and natural extracts is presented in **Tables 3**-**5**. The various combinations of NPK doses with natural extracts applied had significantly different effects on vegetative traits. Plants fertilized with the 75% NPK dose and sprayed with 3 g L^-1^ ADY + 1.5 ml L^-1^ SW had significantly higher values of PH (57.61 and 58.50 cm), NB (17.67 and 18.00 branches/plant), FWR (47.00 and 49.00 g/plant), and DWR (14.77 and 15.13 g/plant) in the two seasons, respectively, compared with the other used combinations. In contrast, the highest significant values of PSD (0.69 and 0.73 cm), AL (55.15 and 55.25 cm^2^), CI (30.20 and 31.20 SPAD units), FWS (111.33 and 114.00 g/plant), and DWS (28.27 and 30.21 g/plant) during both seasons, respectively, were obtained with the combination of 3 g L^-1^ ADY + 1.5 mL L^-1^ SW + 0.5 g L^-1^ GT and the 75% NPK dose compared with the other combinations of NPK doses and natural extracts. In contrast, plants that were not treated with any extract but received the 50% NPK dose had significantly lower values (*P* ≤ 0.05) for all vegetative growth parameters compared with the other treatment combination in the two seasons.

### 3.2 Leaf RWC

Data in **Table 6** reveal that the effects of the 75% and 100% NPK doses on RWC% were similar (*P* ≤ 0.05). Plants fertilized with 100% NPK had the highest RWC values (61.58 and 61.97%), followed by those receiving 75% NPK (61.54 and 61.92%) and then 50% NPK (60.88 and 61. 34%) in the two seasons, respectively. Similarly, application of natural extracts caused notable increases in RWC% relative to the untreated control. Leaves of plants sprayed with 3 g L^-1^ ADY + 0.5 g L^-1^GT and 1.5 ml L^-1^SW + 0.5 g L^-1^ GT had significantly higher RWC values than those of other treatments, with the same significance level (*P* ≤ 0.05). RWC under these treatments reached 63.01 and 63.19%, respectively, compared with 57.53% for the control in the first season, and 63.45 and 63.82%, respectively, compared with 57.76% for the control in the second season. Regarding the interaction effect of the various combinations of NPK doses and natural extracts, data in **Table 6** show that RWC% was not significantly affected by the different treatment combinations in the first season. However, in the second season, the differences among the applied treatments were statistically significant (*P* ≤ 0.05) in the majority of cases. Overall, the highest RWC%, 64.30 and 64.56%, were recorded in plants fertilized with 75% NPK and sprayed with 1.5 mL L^-1^SW + 0.5 g L^-1^ GT for both seasons, respectively. On the other hand, the lowest RWC%, 57.06 and 57.47%, were recorded in plants that received 100% NPK without any natural extract in the two seasons.

**Table 6:** Relative water content and total carbohydrate percentages of *D. repens* as affected by NPK doses, natural extracts, and their interaction in the 2023 and 2024 seasons.

NPK doses (g/plant)	Natural extracts	RWC%		Total carbohydrates %	
		1^st^ season	2^nd^ season	1^st^ season	2^nd^ season
50% NPK		60.88 ± 0.29 B	61.34 ± 0.58 B	8.59 ± 0.08 C	9.25 ± 0.20 C
75% NPK		61.54 ± 0.17 A	61.92 ± 0.35 A	13.46 ± 0.40 A	13.93 ± 0.69 A
100% NPK		61.58 ± 0.18 A	61.97 ± 0.42 A	10.03 ± 0.02 B	10.45 ± 0.34 B
	Control	57.53 ± 0.29 C	57.76 ± 0.45 C	8.36 ± 0.31 E	9.97 ± 0.20 E
	3g L^-1^ADY+0.5g L^-1^GT	63.01 ± 0.01 A	63.45 ± 0.25 A	10.22 ± 0.78 C	10.89 ± 0.39 C
	3g L^-1^ADY+1.5mlL^-1^SW	61.35 ± 0.33 B	61.81 ± 0.44 B	9.68 ± 0.59 D	10.19 ± 0.29 D
	1.5mlL^-1^SW +0.5g L^-1^GT	63.19 ± 0.43 A	63.82 ± 0.78 A	11.75 ± 0.61 B	12.99 ± 0.51 B
	3g L^-1^ADY +1.5mlL^-1^SW +0.5g L^-1^GT	61.35 ± 0.44 B	61.88 ± 0.33 B	13.44 ± 0.76 A	13.45 ± 0.66 A
50% NPK		*ns*	**	**	**
	Control	57.61 ± 0.57 a	57.86 ± 0.52 h	6.05 ± 0.20 o	6.23 ± 0.10 m
	3g L^-1^ADY+0.5g L^-1^GT	62.77± 0.50 a	63.21 ± 0.38 b-e	9.50 ± 0.81 k	10.20 ± 0.40 i
	3g L^-1^ADY+1.5mlL^-1^SW	59.88 ± 0.48 a	60.29 ± 0.24 g	8.60 ± 1.67 m	10.05 ± 0.03 j
	1.5mlL^-1^SW +0.5g L^-1^GT	62.41 ± 1.17 a	63.29 ± 1.74 bcd	7.78 ± 1.06 n	9.15 ± 0.26 k
75% NPK	3g L^-1^ADY +1.5mlL^-1^SW +0.5g L^-1^GT	61.74 ± 0.36 a	62.06 ± 0.18 ef	11.00 ± 0.12 e	10.63 ± 0.27 g
	Control	57.90 ± 0.59 a	57.96 ± 0.58 h	10.35 ± 0.14 g	10.48 ± 0.07 h
	3g L^-1^ADY+0.5g L^-1^GT	62.71± 0.30 a	62.97 ± 0.14 c-f	11.10 ± 0.64 d	11.65 ± 0.32 c
	3g L^-1^ADY+1.5mlL^-1^SW	61.61 ± 0.97 a	62.39 ± 0.56 def	10.45 ± 0.03 f	10.48 ± 0.01 h
	1.5mlL^-1^SW +0.5g L^-1^GT	64.30 ± 0.27 a	64.54 ± 0.13 a	17.33 ± 1.75 b	18.40 ± 1.39 b
100% NPK	3g L^-1^ADY +1.5mlL^-1^SW +0.5g L^-1^GT	61.19 ± 0.68 a	61.78 ± 0.34 f	18.08 ± 1.75 a	18.63 ± 1.66 a
	Control	57.06 ± 0.47 a	57.47 ± 0.24 h	8.70 ± 0.52 l	8.83 ± 0.22 l
	3g L^-1^ADY+0.5g L^-1^GT	63.53 ± 0.75 a	64.19 ± 0.24 ab	10.05 ± 0.89 i	10.83 ± 0.45 f
	3g L^-1^ADY+1.5mlL^-1^SW	62.58 ± 0.54 a	63.76 ± 0.51 abc	10.00 ± 0.06 j	10.05 ± 0.84 j
	1.5mlL^-1^SW +0.5g L^-1^GT	62.86 ± 0.91 a	63.64 ± 0.46 abc	10.15 ± 0.55 h	11.43 ± 0.10 d
	3g L^-1^ADY +1.5mlL^-1^SW +0.5g L^-1^GT	61.85 ± 0.49 a	61.79 ± 0.49 f	11.25 ± 0.20 c	11.10 ± 0.06 e

Means in a column with similar letters are not significantly different (*P* ≤ 0.05) according to DMRT.

ADY, Active dry yeast; SW, seaweed; GT, green tea.

Highly significant = **; significant = *; non-significant = ns.

### 3.3 Leaf total carbohydrates, N, P, and K percentages

Data in **Table 6** show that the 75% NPK dose resulted in significantly higher TC_S_ (13.46 and 13.93%), followed by the 100% NPK dose (10.03 and 10.45%) and, ultimately, the 50% NPK dose (8.59 and 9.25%) in the two seasons, respectively. High N, P, and K percentages (**Table 7**) resulted from the 75% and 100% NPK doses, with no significant differences between these doses (*P* ≤ 0.05) in both seasons. Therefore, the leaves of plants that received 75% NPK contained N (2.82 and 2.86%), P (0.53 and 0.55%), and K (1,63 and 1.91%), while the 100% NPK dose resulted in N (2.78 and 2.85%), P (0.35 and 0.55%), and K (1.64 and 1.92%) in the two seasons, respectively. In addition, spraying with the various natural extracts induced distinct increases in TC_S_, N, P, and K percentages relative to the respective control in both seasons (**Tables 6**, **7**). Notably, plants sprayed with 3 g L^-1^ ADY + 1.5 ml L^-1^SW + 0.5 g L^-1^ GT had the highest TC_S_ (13.44 and 13.45%), N (2.80 and 2.88%), P (0.67 and 0.70%), and K (2.11 and 2.51%) compared with the other natural extract treatments in both seasons. In contrast, leaves of untreated control plants contained lower TC_S_ (8.36 and 9.97%), N (2.61 and 2.71), P (0.40 and 0.35%), and K (1.17 and 1.23%) in the two seasons, respectively. Clearly, the various combinations of NPK doses and natural extracts affected the leaf chemical composition of *D. repens* compared with NPK doses alone (**Tables 6**, **7**). High contents of TC_S_, N, and K, 18.08, 2.85, and 2.18%, respectively, in the first season, and 18.63, 2.97, and 2.69%, respectively, in the second season, resulted from spraying plants with 3 g L^-1^ ADY + 1.5 ml L^-1^SW + 0.5 g L^-1^ GT and fertilizing them with the 75% NPK dose. In contrast, the combination of 50% NPK with 3 g L^-1^ ADY + 1.5 ml L^-1^SW + 0.5 g L^-1^ GT was the most effective treatment for increasing P percentage, with values of 0.70% and 0.74% in the two seasons, respectively. On the other hand, leaves of plants fertilized with 50% NPK dose and untreated with any natural extract contained lower values of TC_S_ (6.05 and 6.23%) (**Table 6**), N (2.56 and 2.60%), P (0.27 and 0.28%), and K (1.02 and 1.09%) (**Table 7**) in the two seasons, respectively.

**Table 7:** Nitrogen, phosphorus, and potassium percentages of *D. repens* as affected by NPK doses, natural extracts, and their interaction in the 2023 and 2024 seasons.

NPK doses (g/plant)	Natural extracts	N%		P%		K%	
		1^st^ season	2^nd^ season	1^st^ season	2^nd^ season	1^st^ season	2^nd^ season
50% NPK		2.66 ± 0.06 B	2.73 ± 0.04 B	0.45 ± 0.02 B	0.48 ± 0.01 B	1.42 ± 0.09 B	1.68 ± 0.03 B
75% NPK		2.82 ± 0.03 A	2.86 ± 0.01 A	0.53 ± 0.01 A	0.55 ± 0.00 A	1.63 ± 0.09 A	1.91 ± 0.04 A
100% NPK		2.78 ± 0.02 A	2.85 ± 0.04 A	0.53 ± 0.01 A	0.55 ± 0.01A	1.64 ± 0.04 A	1.92 ± 0.01 A
	Control	2.61 ± 0.09 B	2.71 ± 0.02 C	0.40 ± 0.01 D	0.35 ± 0.01 D	1.17 ± 0.07 D	1.23 ± 0.03 E
	3g L^-1^ADY+0.5g L^-1^GT	2.79 ± 0.03 A	2.81 ± 0.02 B	0.53 ± 0.02 B	0.55 ± 0.00 B	1.74 ± 0.09 B	1.85 ± 0.06 C
	3g L^-1^ADY+1.5mlL^-1^SW	2.75 ± 0.03 A	2.80 ± 0.03 B	0.53 ± 0.01 B	0.56 ± 0.01 B	1.42 ± 0.09 C	2.14 ± 0.02 B
	1.5mlL^-1^SW +0.5g L^-1^GT	2.78 ± 0.11 A	2.87 ± 0.05 A	0.46 ± 0.02 C	0.48 ± 0.00 C	1.37 ± 0.07 C	1.46 ± 0.04 D
	3g L^-1^ADY +1.5mlL^-1^SW +0.5g L^-1^GT	2.80 ± 0.09 A	2.88 ± 0.02 A	0.67 ± 0.02 A	0.70 ± 0.01 A	2.11 ± 0.03 A	2.51 ± 0.02 A
50% NPK		ns	**	*	**	*	**
	Control	2.50 ± 0.12 a	2.60 ± 0.06 g	0.27 ± 0.01 f	0.28 ± 0.01 g	1.02 ± 0.08 g	1.09 ± 0.04 i
	3g L^-1^ADY+0.5g L^-1^GT	2.65 ± 0.03 a	2.65 ± 0.03 fg	0.43 ± 0.02 de	0.44 ± 0.01 e	1.60 ± 0.20 cd	1.78 ± 0.10 f
	3g L^-1^ADY+1.5mlL^-1^SW	2.72 ± 0.07 a	2.79 ± 0.04 de	0.50 ± 0.05 cd	0.54 ± 0.02 cd	1.33 ± 0.18 def	2.05 ± 0.01 cd
	1.5mlL^-1^SW +0.5g L^-1^GT	2.70 ± 0.17 a	2.85 ± 0.09 cd	0.36 ± 0.03 ef	0.38 ± 0.03 f	1.13 ± 0.14 fg	1.11 ± 0.14 i
75% NPK	3g L^-1^ADY +1.5mlL^-1^SW+0.5g L^-1^GT	2.70 ± 0.10 a	2.80 ± 0.03 de	0.70 ± 0.05 a	0.74 ± 0.03 a	2.03 ± 0.02 a	2.41 ± 0.15 b
	Control	2.70 ± 0.10 a	2.80 ± 0.03 de	0.36 ± 0.01 ef	0.37 ± 0.01 f	1.32 ± 0.11 def	1.41 ± 0.05 h
	3g L^-1^ADY+0.5g L^-1^GT	2.90 ± 0.06 a	2.84 ± 0.01 cd	0.59 ± 0.03 bc	0.62 ± 0.02 b	1.70 ± 0.12 bc	1.80 ± 0.06 ef
	3g L^-1^ADY+1.5mlL^-1^SW	2.85 ± 0.03 a	2.87 ± 0.01 bcd	0.54 ± 0.02 bc	0.55 ± 0.02 cd	1.58 ± 0.13 cd	2.25 ± 0.04 bc
	1.5mlL^-1^SW +0.5g L^-1^GT	2.85 ± 0.09 a	2.92 ± 0.04 abc	0.50 ± 0.02 cd	0.52 ± 0.02 d	1.47 ± 0.09 cde	1.55 ± 0.04 gh
100% NPK	3g L^-1^ADY +1.5mlL^-1^SW +0.5g L^-1^GT	2.85 ± 0.03 a	2.97 ± 0.07 a	0.69 ± 0.03 a	0.72 ± 0.01 a	2.18 ± 0.09 a	2.69 ± 0.06 a
	Control	2.65 ± 0.09 a	2.72 ± 0.04 ef	0.40 ± 0.01 e	0.40 ± 0.00 ef	1.18 ± 0.03 efg	1.20 ± 0.01 i
	3g L^-1^ADY+0.5g L^-1^GT	2.82 ± 0.01 a	2.95 ± 0.03 ab	0.57 ± 0.05 bc	0.60 ± 0.03 bc	1.93 ± 0.04 ab	2.00 ± 0.03 de
	3g L^-1^ADY+1.5mlL^-1^SW	2.70 ± 0.06 a	2.75 ± 0.03 e	0.57 ± 0.03 bc	0.60 ± 0.02 bc	1.38 ± 0.07 def	2.11 ± 0.01 cd
	1.5mlL^-1^SW +0.5g L^-1^GT	2.80 ± 0.06 a	2.85 ± 0.03 cd	0.52 ± 0.03 c	0.54 ± 0.03 cd	1.52 ± 0.22 cd	1.73 ± 0.07 fg
	3g L^-1^ADY +1.5mlL^-1^SW +0.5g L^-1^GT	2.85 ± 0.14 a	2.87 ± 0.01 bcd	0.62 ± 0.02 ab	0.64 ± 0.01 b	2.10 ± 0.01 a	2.44 ± 0.04 b

Means with the same letters in a column are not significantly different (*P ≤ 0.05*) according to DMRT.

ADY, Active dry yeast; SW, seaweed; GT, green tea.

Highly significant = **; significant = *; non-significant = ns.

### 3.4 Leaf CAT, POD, and PPO activities

**Figure 1:**
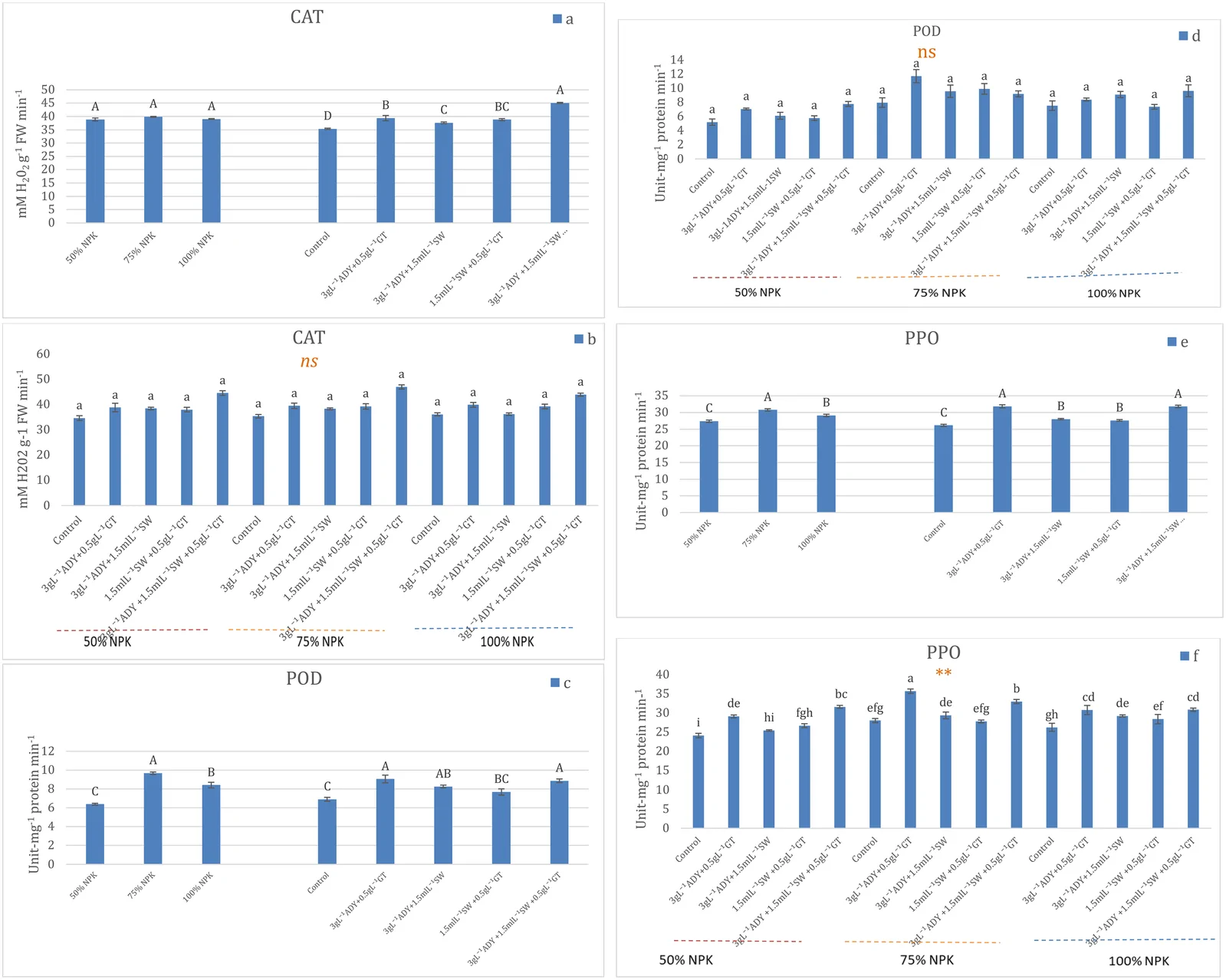
Effect of NPK doses, natural extracts, and their interaction on activates of CAT **(a, b)**, POD **(c, d)**, and PPO **(e, f)**. Within a Figure, means with similar letters are not significantly different (*P* ≤ 0.05) according to DMRT. ADY, Active dry yeast; SW, seaweed; GT, green tea. Highly significant = **; significant = *; non-significant = ns.

**Figures 1a, c, e** shows the influences of NPK doses on CAT, POD, and PPO activities in leaves of *D. repens* in the 2024 season. The 75% NPK dose resulted in the highest CAT, POD, and PPO activities compared with the 50% and 100% NPK doses. This 75% dose resulted in CAT, POD, and PPO activities of 39.85 mM H_2_O_2_ g^-1^FW min^-1^, 9.67 unit-mg^-1^ protein min^-1^, and 30.77 unit-mg^-1^ protein min.^-1^, respectively. This was followed by the 100% NPK dose, which resulted in CAT of 39.04 mM H_2_O_2_ g^-1^ FW min.^-1^, POD of 8.41 unit- mg^-1^ protein min.^-1^, and PPO activities of 29.10 unit-mg^-1^ protein min.^-1^. Finally, the 50% NPK dose resulted in lower CAT, POD, and PPO activities, at 38.86 mM H_2_O_2_ g^-1^ FW min.^-1^, 6.38 unit-mg^-1^ protein min.^-1^, and 27.39 unit-mg^-1^ protein min^-1^, respectively. Plants sprayed with natural extracts resulted in higher values in comparison to unsprayed plants of control (**Figures 1a, c, e**). Clearly, the treatment of 3 g L^-1^ ADY + 1.5 ml L^-1^SW + 0.5 g L^-1^ GT resulted in higher CAT activity (45.10 mM H_2_O_2_ g^-1^ FW min.^-1^). In contrast, application of 3 g L^-1^ ADY + 0.5 g L^-1^ GT and 3 g L^-1^ ADY + 1.5 ml L^-1^SW + 0.5 g L^-1^ GT treatments resulted in the highest significant POD activities, 9.06 and 8.87 unit-mg^-1^ protein min.^-1^ and PPO activities, 31.84 and 31.80 unit-mg^-1^ protein min.^-1^, respectively, with the same significance level (*P ≤ 0.05*). By contrast, CAT, POD, and PPO activities in leaves of control plants were 35.35 mM H_2_O_2_ g^-1^ FW min^-1^, 6.90 unit-mg^-1^ protein min^-1^, and 26.14 unit-mg^-1^ protein min^-1^, respectively, which were lower than those observed with the other natural extract treatments.

Data in **Figures 1b, d, f** showed that all combinations of NPK doses and biostimulant extracts had non-significant effects on CAT and POD activities, whereas their impacts on PPO were significant. The highest and lowest CAT activities resulted from the combined 75% NPK dose with 3 g L^-1^ ADY + 1.5 ml L^-1^SW + 0.5 g L^-1^ GT and 50% NPK without spraying of any extract, with values of 46.93 and 34.63 mM H_2_O_2_ g^-1^ FW min^-1^, respectively. Plants fertilized with 75% NPK and sprayed with 3 g L^-1^ ADY + 0.5 g L^-1^ GT had the highest POD and PPO activities in their leaves, at 11.70 and 35.67 unit-mg^-1^ protein min^-1^, respectively. In contrast, plants fertilized with 50% NPK without spraying with biostimulant extracts had the lowest POD and PPO activities, 5.20 and 24.13 unit-mg^-1^ protein min^-1^, respectively.

### 3.5 TPCs and AOA

**Figure 2:**
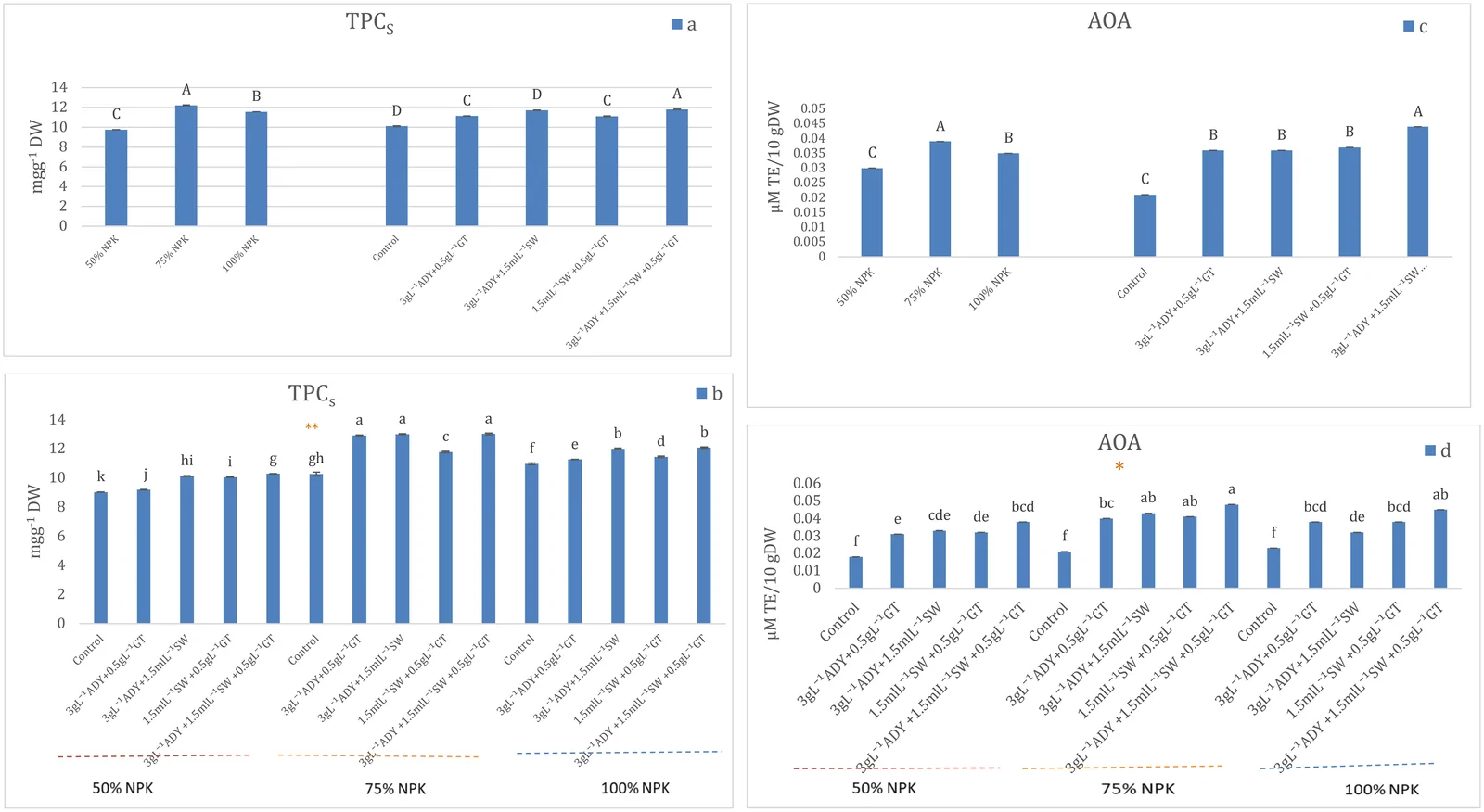
Effect of NPK doses, natural extracts, and their interaction on the content of TPC_S_ **(a, b)**, and AOA **(c, d)**. Means with the same letters are not significantly different (*P* ≤ 0.05) according to DMRT. ADY, Active dry yeast; SW, seaweed; GT, green tea. Highly significant = **; significant = *; non-significant = ns.

Data in **Figures 2a, c** revealed that the 75% NPK dose achieved the highest significant TPC_S_ and AOA values, at 12.21 mg g^-1^DW and 0,039 μM TE 10 g^-1^ DW, respectively. This was followed by the 100% NPK dose, which resulted in TPC_S_ and AOA values of 11.56 mg g^-1^DW and 0.035 μM TE 10 g^-1^ DW, respectively. The 50% NPK dose resulted in the least significant TPC_S_ and AOA contents, at 9.75 mg g^-1^ DW and 0.030 μM TE 10 g^-1^ DW, respectively. Similarly, the leaves of plants sprayed with 3 g L^-1^ ADY + 1.5 ml L^-1^SW + 0.5 g L^-1^ GT contained higher significant values of TPC_S_ (11.81 mg g^-1^ DW) and AOA (0.044 μM TE 10 g^-1^ DW) values (**Figures 2a, c**). Conversely, the leaves of unsprayed control plants contained lower significant TPC_S_ (10.10 mg g^-1^ DW) and AOA (0.021 μM TE 10 g^-1^ DW) values. Concerning the interaction effect between NPK doses and natural extracts, data presented in **Figures 2b, d** showed that the 75% NPK dose combined with the treatments of 3 g L^-1^ ADY + 1.5 ml L^-1^SW + 0.5 g L^-1^ GT, 3 g L^-1^ ADY + 1.5 ml L^-1^SW, and 3 g L^-1^ ADY + 0.5 g L^-1^ GT resulted in the highest significant TPC_S_ values of 13.03, 13.02, and 12.93 mg g^-1^ DW, respectively, with the same significance level (*P ≤ 0.05*). In addition, the 75% NPK and the 100% NPK doses combined with 3 g L^-1^ ADY + 1.5 ml L^-1^SW + 0.5 g L^-1^ GT produced significantly higher AOA values of 0.048 and 0.045 μM TE 10 g^-1^ DW, respectively, with no significant difference between the two treatments. By contrast, the leaves of plants fertilized with the 50% NPK dose without any natural extract treatment contained lower significant TPC_S_ (9.04 mg g^-1^DW) and AOA (0.018 μM TE 10 g^-1^ DW) values compared with the other combinations (**Figures 2b, d**).

## 4 Discussion

### 4.1 Impact of NPK doses

Fertilization is applied to fulfill plant requirements for essential nutrients. Appropriate application of N, P, and K fertilizers can effectively stimulate vegetative growth and improve plant structure (Nemadodzi et al., 2017; Xing et al., 2023). Fertilizer suitability can influence soil fertility and, consequently, plant performance, indicating a close relationship among soil properties, nutrient availability, and plant growth (Chen et al., 2019; Zhang et al., 2023).

The results of the present study demonstrated that the most suitable NPK dose for achieving the best values of the majority of studied traits was 75% of the recommended NPK dose, except for PSD and RWC%, where the 100% NPK dose produced the highest values. These findings suggest that the full recommended NPK dose may exceed the nutritional requirements of *Duranta repens* for N, P, and K. Moreover, excessive application of chemical fertilizers can degrade soil quality, eutrophicate water bodies, and contaminate air and groundwater, and overuse of chemical fertilizers can also cause inefficient nutrient utilization and habitat disruption, posing several challenges to sustainable agriculture (Congreves and Van Eerd, 2015). Excessive fertilizer application may lead to disease incidence, reduced leaf and root development, and nitrate accumulation in Chinese cabbage (Shi et al., 2021). Furthermore, overuse of fertilizers can cause soil acidification and severe nutrient imbalance, which ultimately affects plant productivity and economic efficiency (Raliya et al., 2017; Xue et al., 2020).

The beneficial effects of N, P, and K fertilizers are attributed to their essential roles in plant physiological processes, including stimulation of enzyme activities, enhancement of carbohydrate accumulation through photosynthesis, and promotion of cell division and elongation. In addition, the application of NPK fertilizers can increase indole-3-acetic acid (IAA) levels and improve photosynthetic efficiency, thereby enhancing carbohydrate production (Alkhafaji and Khalil, 2019), which subsequently improves the studied characteristics of *Citrus limon*. Moreover, NPK elements contribute to meristematic growth, energy transfer, chlorophyll biosynthesis, and the stimulation of enzymes and various compounds such as amino acids, nucleic acids, amides, proteins, and sugars, owing to their fundamental roles in protoplasm formation and biosynthetic processes within plant cells (Al-Marsoumi and Al-Hadethi, 2020; Al-Khafaji et al., 2022). Additionally, Choudhary et al. (2022) and Zhang et al. (2022) documented that appropriate availability and balance of NPK nutrients are closely associated with enhanced N uptake, which, in turn, improves mesophyll cell and chloroplast development and increases chlorophyll stability, thereby elevating chlorophyll content in plants. In practical agricultural production, the proportions of N, P, and K fertilizers are often unbalanced because of the lack of scientifically based fertilization ratios. This imbalance negatively affects nutrient uptake and utilization by plants, leading to reductions in yield and quality and increasing the risks of nutrient deficiency and environmental contamination (Rahman and Zhang, 2018). The vegetative characteristics of *Datura stramonium*, including PH, number of leaves, total leaf area per plant, and fresh and dry weights of leaves per plant, were significantly increased by the application of 100% NPK fertilization (120 kg urea + 120 kg ammonium sulfate + 100 kg calcium superphosphate + 50 kg K sulfate per feddan) compared with 25% or 50% NPK treatments (Nassar et al., 2015). Previously, Gad (2019) reported that the application of 10 g plant^−1^ of NPK (17:17:17) was the most effective treatment for increasing leaf number, total leaf area per plant, and fresh and dry weights of the aerial parts of *Bougainvillea glabra* cv. Snow White. However, the highest values of PH, number of shoots per plant, leaf N, P, and K contents, carbohydrate content, chlorophyll concentration, and flower bract characteristics were obtained with 20 g NPK plant^−1^. The present findings are also consistent with those of Nofal et al. (2021) on *Tagetes erecta* fertilized with 2 g NPK plant^−1^ applied three times, where leaf chlorophyll a and b contents, N, P, and K levels, TCs, and total phenols were significantly enhanced compared with untreated plants. According to Jiang et al. (2024), the highest values of PH, stem diameter, and leaf area during the seedling stage of C. morifolium were obtained with the combined application of 330 kg ha^−1^ N, 196 kg ha^−1^ P_2_O_5_, and 450 kg ha^−1^ K_2_O. During the branching and flowering stages, the greatest increases in these parameters, along with chlorophyll content, were achieved with 330 kg ha^−1^ N, 196 kg ha^−1^ P_2_O_5_, and 300 kg ha^−1^ K_2_O. In addition, Jawad and Khalil (2024) found that leaf dry weight and chlorophyll content of rose plants increased following the application of NPK at 5 g L^−1^. These findings are further supported by the study of Liu et al. (2024) on *Sapindas mukorossi*, who observed that leaf area index and leaf contents of soluble sugars, starch, N, P, K, and chlorophyll were significantly enhanced by various NPK combinations. Our findings suggested a non-significant enhancement in CAT and significant enhancements in POD and PPO activities and TPC_S_ and AOA contents after application of the 75% NPK dose compared with the 50% NPK or 100% NPK doses. These findings indicate that 75% of the recommended NPK dose is suitable to supply *D. repens* its requirements for N, P, and K. Particularly, the plants grown in the open field may have been exposed to undesirable conditions, such as high temperature (approximately 30 °C), insects, or lower (50% NPK) or excessive (100% NPK) fertilization (Zhang et al., 2020). Furthermore, plant defense systems activate enzymes, such as PPO, POD, CAT, and superoxide dismutase (SOD), which contribute to disease resistance and protection against ROS toxicity. Plants also produce phenylalanine ammonia-lyase (PAL), a defense-related enzyme that plays an important role in these protective mechanisms (Güneş et al., 2019; Sachdev et al., 2021).

Moreover, total phenolic compounds (TPCs) and AOA play important roles in plant protection against unfavorable environmental conditions. Phenolic compounds contain hydroxyl groups that contribute significantly to ROS scavenging; therefore, TPCs are often directly associated with AOA in plants (Tosun et al., 2009). In addition, TPCs exhibit antioxidant properties and participate in enzymatic activity and primary metabolic processes (Jokar and Ronaghi, 2015). Several studies have demonstrated a positive relationship between TPCs and AOA in plants such as sunflower (Kiarostami et al., 2010), rosemary (Rady et al., 2011), lavender (Chrysargyris et al., 2018), sweet basil (Ahmed et al., 2019; El-Mahrouk et al., 2024a), and sage (El-Mahrouk et al., 2024b). Gaaliche et al. (2019) reported that foliar spraying of Bouhouli trees with K_2_SO_4_ induced noticeable changes in TPCs, flavonoids, and chlorogenic acid content compared with untreated control plants. Likewise, Jiang et al. (2024) verified that the application of 330 kg ha^−1^ N + 196 kg ha^−1^ P_2_O_5_ + 450 kg ha^−1^ K_2_O and 330 kg ha^−1^ N + 196 kg ha^−1^ P_2_O_5_ + 300 kg ha^−1^ K_2_O increased PAL and POD activities, respectively, in C. morifolium flowers.

### 4.2 Impact of ADY, SW, and GT extracts

The current study demonstrated the distinct positive effects of applying ADY, SW, and GT extracts on the investigated traits. Foliar application represents an effective method for utilizing these extracts because they can be readily absorbed through plant leaves and subsequently translocated to other plant parts. This process contributes to physiological regulation and helps alleviate the adverse effects of unfavorable environmental conditions. Previous studies have highlighted the beneficial effects of natural extracts on various physiological and biochemical processes within plant cells. ADY has been reported to increase proline accumulation and antioxidant enzyme activities while alleviating oxidative stress (Esmaeilzadeh Bahabadi and Sharifi, 2013), and to reduce lipid peroxidation (Alzandi and Naguib, 2022). The present results suggest that the application of ADY plays an important role in enhancing growth characteristics (**Tables 3**–**5**) and the chemical (**Tables 6**, **7**) and biochemical (**Figures 1**, **2**) composition of the leaves of *D. repens*, owing to its richness in macro- and micronutrients, vitamins, proteins, hormones, lipids, amino acids, and carbohydrates (Amer, 2004; Ghoname et al., 2009; Tao et al., 2022). El-Shawa et al. (2020) demonstrated that foliar spraying of calendula plants with ADY at 4, 8, and 12 g L^−1^ significantly enhanced vegetative and floral traits, total chlorophyll content, N, P, and K contents, RWC%, and the activities of PPO, POD, CAT, and SOD enzymes compared with untreated controls. Similarly, Khudair and Hajam (2021) reported that the highest vegetative, floral, and root characteristics of Chinese carnation were achieved with the application of 2 or 4 mL L^−1−1^ Algidex as an organic fertilizer. In addition, Gholami et al. (2023) concluded that vegetative growth parameters, total chlorophyll, sugars, phenols, carbohydrates, and the activities of SOD, PPO, and CAT in cowpea were significantly improved by the application of 12 g L^−1^ ADY. They also found that the combination of 24-epibrassinolide (10 μM) with 12 g L^−1^ ADY was the most effective treatment for enhancing seed yield, pod weight, and chlorophyll content in this plant species.

Numerous studies have reported the significant positive effects of SW extracts on vegetative growth performance and the chemical and biochemical composition of plants, resulting in improvements in various physiological and biochemical processes within plant cells. This aligns with our results. These beneficial effects are attributed to the rich composition of SW extracts, which contain essential nutrients, amino acids, hormones, proteins, fatty acids, and polysaccharides (Craigie, 2011; Reddy and Saravanan, 2013; Yakhin et al., 2017; Ali et al., 2021; Jafarlou Bahmani et al., 2021). Furthermore, SW extracts applied either as foliar sprays or to the rhizosphere have been shown to enhance plant productivity and quality by regulating nutrient absorption and improving nutrient use efficiency, while also increasing plant tolerance to both abiotic and biotic stresses (Sharma et al., 2014; Koleška et al., 2017; Rouphael and Colla, 2018). Studies conducted on rose plants (Kularathne et al., 2021), *Lantana camara* and *Abelia × grandiflora* shrubs (Loconsole et al., 2022), *Citrullus lanatus* (Radwan et al., 2023), kiwifruit (Rana et al., 2023), and strawberry (Bagh et al., 2024) demonstrated that foliar spraying with SW extracts significantly improved vegetative growth and chemical and biochemical characteristics. This concept was further supported by Mohamed et al. (2021), who found that sweet pepper plants treated with 3 g L^−1^ ADY as a soil application combined with 3 g L^−1^ SW extract as a foliar spray exhibited improved PH, leaf area, and fresh and dry weights. In contrast, the highest carbohydrate, P, and K contents in fruits were obtained following the application of SW extract at 3 g L^−1^ combined with folic acid. Moreover, the authors concluded that the integration of effective microorganisms with SW and ADY extracts enhanced plant growth, nutrient content, enzymatic activities, fruit yield, and the quality and nutritional value of sweet pepper fruits, while also contributing to environmental sustainability by reducing contamination. Similarly, uz Zaman et al. (2025) reported that foliar spraying with SW extract at 1 or 3 g L^−1^ significantly increased growth and biomass production in hemp compared with untreated controls. In addition, the synergistic effects of SW extract effectively reduced H_2_O_2_ production, proline accumulation, and malondialdehyde generation, while enhancing soluble sugar and protein contents. Moreover, the present study highlighted the role of GT as a biostimulant in improving the growth and related characteristics of *D. repens*, particularly because no previous studies have investigated the effects of GT on this species. The observed improvement in *D. repens* parameters following GT application may be attributed to its content of caffeine, volatile oils, tannins, and polyphenols, which possess stimulatory properties. In addition, GT contains vitamins and mineral elements and can enhance enzymatic activities (Armstrong et al., 2020). Furthermore, the application of GT may protect plant cells from senescence and promote vegetative growth because it contains antioxidant compounds such as catechin and tocopherol, which play important roles in protecting plant cells against ROS that can damage DNA, proteins, and lipids (Pastoriza et al., 2017; Ali et al., 2022). Ahmed et al. (2014) reported that different concentrations of GT significantly increased leaf area and leaf dry weight in Keitte mango plants. Similarly, *Allium cepa* seedlings treated with 50 mg L^−1^ GT leaf extract exhibited enhanced seedling growth (Çavuşoğlu, 2020). This finding was further supported by Abdel Aal et al. (2021), who demonstrated that GT extract at concentrations ranging from 0.05% to 2% improved growth characteristics and nutrient concentrations in Barhee date palm plants. In addition, Moustafa (2020) showed that foliar application of GT extract at concentrations ranging from 0.01% to 0.8% significantly increased PH, number of leaves and branches per plant, stem diameter, leaf area, and fresh and dry weights of stems, leaves, and roots in aralia plants. The treatment also enhanced leaf N, P, and K percentages, along with chlorophyll a, chlorophyll b, and total chlorophyll contents compared with untreated plants. Furthermore, the Zibo and *Camencita castor* bean varieties achieved the highest leaf dry weight and fruit number per plant following treatment with 75 mL L^−1^ GT extract (Hassan and Ibrahim, 2024).

### 4.3 Impact of combinations of NPK doses and natural extracts

The present data demonstrated that the application of different combinations of NPK fertilizers and natural extracts resulted in pronounced increases in the studied parameters compared with the application of NPK fertilizers alone. Among the tested treatments, the combination of 75% NPK with 3 g L^−1^ ADY, 1.5 mL L^−1^ SW, and 0.5 g L^−1^ GT was the most effective, producing the highest values of the investigated parameters, with few exceptions (PH, NB, RFW, and RDW with 75% NPK + 3 g L^-1^ ADY + 1.5 m L^−1^ SW; RWC% with 75% NPK + 1.5 m L^−1^ SW + 0.5 g L^−1^ GT; P% with 50% NPK + 3 g L^-1^ ADY + 1.5 m L^−1^ SW + 0.5 g L^−1^ GT; and POD and PPO with 75% NPK + 3 g L^-1^ ADY + 0.5 g L^−1^ GT). These findings suggest that the application of 75% NPK combined with 3 g L^−1^ ADY + 1.5 mL L^−1^ SW + 0.5 g L^−1^ GT provided sufficient macro- and micronutrients, in addition to the stimulatory compounds present in ADY, SW, and GT extracts. Collectively, these components contribute to amino acid and protein synthesis, enhancement of photosynthesis, accumulation of carbohydrates, production of energy compounds, stimulation of enzymatic activities, and alleviation of the harmful effects of ROS, ultimately resulting in improved plant growth and development. Our results clearly indicate that 50%NPK combined with natural extracts did not provide sufficient nutrients to meet the essential nutrient requirements of *D. repens*, whereas the 100% NPK dose combined with natural extracts may have provided nutrients in excess of the requirements of *D. repens*. The present results are consistent with those of Teja et al. (2017), who concluded that N and K are essential components in chlorophyll, protein, and amino acid synthesis. Therefore, increased N and K levels can enhance chlorophyll content and stimulate photosynthesis, leading to improved growth of *Chrysanthemum coronarium*. Similarly, El-Azzony et al. (2018) reported that 75% of the recommended NPK level combined with 100 mL effective microorganisms per shrub was the most effective treatment for improving the vegetative growth of *Jatropha curcas* when compared to 100% NPK dose + 100 mL effective microorganisms. These findings are further supported by Al-Hamzawi (2019), who demonstrated that SW extract combined with 1or 2 mL L^−1^ micronutrients significantly enhanced the vegetative growth and flowering characteristics of *Dianthus chinensis* and *Gazania rigens*. Furthermore, sage plants treated with 75% of the recommended NPK dose combined with biofertilizers (*Azotobacter chroococcum*, *Bacillus megaterium* var. phosphaticum, and *Bacillus cereus*) produced significantly taller plants, greater numbers of branches, higher fresh and dry weights per plant, and increased contents of carbohydrates, antioxidants, chlorophyll, and total phenols compared with 100% NPK + biofertilizers (Amer et al., 2019).

Similarly, Abdelaal et al. (2021) reported that the combination of 8 g L^−1^ ADY with 300 mM chitosan increased PH, chlorophyll a and b contents, and RWC, while alleviating ROS accumulation and regulating antioxidant enzyme activities in stressed garlic plants. In addition, Nofal et al. (2021) documented that the greatest enhancement in leaf N, P, and K contents, chlorophyll a and b, carotenoids, total indoles, TCs, and total phenols of *Tagetes erecta* var. dwarf chrysanthemum was achieved following the application of 2 g L^−1^ SW extract combined with 2 g pot^−1^ NPK fertilizer. Additionally, Abdulrazzaq and Abdulmajeed (2025) found that 0.5 g L^-1^ NPK + 0.5 g L^-1^ SW resulted in higher values of leaf fresh weight, bract numbers, and carotene content in Bougainvillea Dwarf Pink compared with 1 g L^-1^ NPK + 1 g L^-1^ SW.

Likewise, Faizah et al. (2025) demonstrated that the application of 10 g plant^−1^ NPK combined with 12 mL L^−1^ liquid organic fertilizer produced the highest growth and yield parameters in bean plants. Furthermore, Bharathkumar et al. (2025), working on *China aster* cv. “Azka × Kamini”, showed that the treatment consisting of 75% NPK combined with Azotobacter, phosphate-solubilizing bacteria (PSB), K-solubilizing bacteria (KSB), and 0.5% SW extract reduced mortality rate and increased survival percentage, seedling height, and vigor indices. Soliman et al. (2026) reported that application of biochar at 12.5 tons ha^-1^ combined with 5 g L^-1^ NPK as foliar spraying and 4 mL L^-1^ SW extract resulted in the highest biomass accumulation, chlorophyll content, and nutrient uptake (N, P, and K) of spearmint grown in sandy soil compared with 12.5 tons ha^-1^ biochar, 12.5 tons ha^-1^ biochar + 5 g L^-1^ NPK, and 12.5 tons ha^-1^ + 5 g L^-1^ NPK + 2 mL SW extract treatment. Overall, the present results suggest that *Duranta repens* can be effectively fertilized with 75% of the recommended NPK dose, combined with 3 g L^−1^ ADY, 1.5 mL L^−1^ SW, and 0.5 g L^−1^ GT.

## 5 Conclusion

The present study highlights the potential of combining NPK fertilizers with ADY, SW, and GT extracts to enhance the overall growth performance and the chemical and biochemical composition of *Duranta repens*. The use of natural extracts as partial substitutes for chemical fertilizers appears promising because of their positive effects on physiological, chemical, and biochemical processes within plant cells, including the promotion of cell division and elongation. Collectively, these effects contribute to improved plant growth characteristics. The findings of the current study demonstrated that the application of 75% of the recommended NPK dose combined with 3 g L^−1^ ADY + 1.5 mL L^−1^ SW + 0.5 g L^−1^ GT was the most suitable treatment for enhancing the vegetative, chemical, and biochemical parameters of *D. repens* intended for landscape applications and medicinal uses. Moreover, this fertilization strategy may contribute to reducing environmental pollution associated with the excessive use of chemical fertilizers.

Further studies are recommended to investigate fertilization programs for ornamental trees and shrubs at different growth stages, using combinations of chemical fertilizers and natural extracts, to determine the most effective fertilization strategy for each plant species.

## Data Availability

The original contributions presented in the study are included in the article/supplementary material. Further inquiries can be directed to the corresponding authors.
